# Fatigue Driving Detection Method Based on Combination of BP Neural Network and Time Cumulative Effect

**DOI:** 10.3390/s22134717

**Published:** 2022-06-22

**Authors:** Jian Chen, Ming Yan, Feng Zhu, Jing Xu, Hai Li, Xiaoguang Sun

**Affiliations:** School of Mechanical Engineering, Yangzhou University, Yangzhou 225127, China; jian.chen@yzu.edu.cn (J.C.); mingyan.yzu@hotmail.com (M.Y.); fengzhu.yzu@hotmail.com (F.Z.); jingxu@yzu.edu.cn (J.X.); hai.li200888@gmail.com (H.L.)

**Keywords:** fatigue driving detection, Adaboost algorithm, cascade regression tree, BP neural network algorithm, time accumulation effect

## Abstract

Fatigue driving has always received a lot of attention, but few studies have focused on the fact that human fatigue is a cumulative process over time, and there are no models available to reflect this phenomenon. Furthermore, the problem of incorrect detection due to facial expression is still not well addressed. In this article, a model based on BP neural network and time cumulative effect was proposed to solve these problems. Experimental data were used to carry out this work and validate the proposed method. Firstly, the Adaboost algorithm was applied to detect faces, and the Kalman filter algorithm was used to trace the face movement. Then, a cascade regression tree-based method was used to detect the 68 facial landmarks and an improved method combining key points and image processing was adopted to calculate the eye aspect ratio (EAR). After that, a BP neural network model was developed and trained by selecting three characteristics: the longest period of continuous eye closure, number of yawns, and percentage of eye closure time (PERCLOS), and then the detection results without and with facial expressions were discussed and analyzed. Finally, by introducing the Sigmoid function, a fatigue detection model considering the time accumulation effect was established, and the drivers’ fatigue state was identified segment by segment through the recorded video. Compared with the traditional BP neural network model, the detection accuracies of the proposed model without and with facial expressions increased by 3.3% and 8.4%, respectively. The number of incorrect detections in the awake state also decreased obviously. The experimental results show that the proposed model can effectively filter out incorrect detections caused by facial expressions and truly reflect that driver fatigue is a time accumulating process.

## 1. Introduction

Driver fatigue is one of the major causes of the increasing number of road accidents. It is found that driver fatigue accounts for 35–45% of all vehicle accidents [1]. However, the initial signs of fatigue can be detected before a critical situation arises, and developing systems to automatically detect driver fatigue and advise drivers to take a break in time has received increased interest. There are many methods available to determine the drowsiness state of a driver [1,2,3,4], including detection based on vehicle information, the driver’s information, and multi-information fusion.

The vehicle information-based method mainly identifies fatigue driving by collecting vehicle driving information and analyzing the abnormal data. Related parameters include the vehicle speed, vehicle acceleration, vehicle steering angle, steering wheel grip, and so on. At present, most vehicles are equipped with various sensors to monitor these parameters, and, through data analysis of these parameters, the state of fatigue of drivers can be indirectly detected. The main disadvantage of this method is low accuracy.

Detection methods based on the driver’s information can be divided into two types, one is based on the driver’s physiological information, and the other is based on the driver’s behavior characteristics. When a driver appears fatigued, their physiological indicators will show certain regular changes, which can directly reflect the fatigue state of the driver. The main detection parameters include electroencephalogram (EEG), electrooculogram (EOG), electrocardiogram (ECG), electromyogram (EMG), heart rate variability (HRV), and so on. Such methods can detect fatigue states more accurately, but the need to wear complex equipment makes it very inconvenient [3]. In addition, when a driver enters the fatigue state, their facial behavioral characteristics will change correspondingly. These features include eye movements, mouth state, head posture, and so on. It is an effective method to extract facial features using computer vision and carry out fatigue detection according to these characteristics. For these methods, the establishment of complex models and the large amount of image data to be processed pose new challenges to the computing power of computers.

In many cases, due to the influence of factors such as environment, road conditions, and individual differences, the reliability of using a single fatigue feature to judge the driver’s state is relatively low. The fusion of multiple features can greatly improve the accuracy and robustness of fatigue detection, and it is more suitable for changeable environments. However, it requires various information that is difficult to obtain, and a fusion model is difficult to establish [4].

For the methods mentioned above, the function of fatigue detection has been realized, but few literatures consider that fatigue is a process that gradually deepens over time; no model available can reflect this phenomenon. In addition, for the method based on driver’s behavior characteristics, there is not much discussion about the incorrect detections caused by the dim light, deeper eye sockets, changes in facial expressions, head movements, and so on; despite this, their influence cannot be ignored.

The objective of this work is to build a new method to reflect that fatigue is a process that gradually deepens over time and to eliminate the incorrect detections that may be caused by the factors mentioned above. The main contributions of this paper are as follows: (1) an improved method combination of key points and image processing was used to reduce the excessive eye aspect ratio (EAR) errors; (2) the influence of facial expressions on the detection accuracy of the BP neural network model was discussed and analyzed; (3) a model based on the time accumulation effect was proposed to eliminate the influence of interfering factors, which can better reflect the real fatigue state.

The remainder of this paper is organized as follows: Section 2 presents the previous work on fatigue driving detection. Section 3 introduces the steps of driver fatigue detection in detail and proposes the model based on the time accumulation effect. Section 4 shows the effectiveness and robustness of the proposed model by comparing it with the detection results based on the BP neural network model. Section 5 discusses the obtained results. Section 6 concludes the full text and looks forward to future work.

## 2. Related Work

### 2.1. Detection Based on Vehicle Information

For driving fatigue detection based on vehicle information, Zhang et al. [5] proposed a method based on the characteristics of steering wheel angle; the detection model was established by support vector machines (SVM), and a cross-validation method was introduced to optimize the parameters. Li et al. [6] proposed an unobtrusive way to detect fatigue for drivers through grip forces on the steering wheel, wavelet transformation was introduced to extract fatigue-related features from wavelet coefficients, and the performance of k-nearest neighbors, linear discriminant analysis, and SVM were compared on the task of discriminating drowsy and awake states. An adaptive vigilance estimation methodology based on steering wheel angle information was proposed by He et al. [7], the correlation coefficient between steering wheel angle and lane deviation was computed, and the driver fatigue evaluation model was established by the Bayesian Network (BN).

### 2.2. Detection Based on Driver Information

For fatigue detection based on the driver’s physiological parameters, Papadelis et al. [8] provided convincing examples of using the EEG to develop an automated system and continuously track the fatigue and drowsiness states of the human driver. Jap et al. [9] investigated the changes in EEG activity in train drivers during a monotonous train-driving session; four combinations of EEG activities were compared to investigate the difference in the performance of the corresponding different equations. Lin et al. [10] proposed a novel brain-computer interface (BCI) system that can acquire and analyze EEG signals in real-time to monitor human physiological and cognitive states. The real-time EEG-based drowsiness monitoring and warning algorithms were implemented and integrated into the system to close the loop of the BCI system, which can provide warning signals to the users when needed. In addition, the fusion of different physiological information was also widely investigated [11,12,13,14]. Although these models can accurately recognize fatigue states, electrodes need to contact the skin of the driver for physiological signal detection, which can cause an uncomfortable and annoying feeling. Hence, they are not suitable for fatigue recognition over the long term. Martins et al. [15] provided an overview of the current state-of-the-art monitoring variables associated with fatigue via wearables and detected potential gaps and pitfalls in current knowledge.

For detection based on driver’s behavior characteristics, Dziuda et al. [16] presented a camera-based prototype sensor for detecting fatigue and drowsiness in drivers. The percentage of eye closure time (PERCLOS), eye closure duration (ECD), and frequency of eye closure (FEC) were selected as eye closure-associated fatigue indicators. In the paper of Cui et al. [17], a lightweight neural network model was designed to solve the problem of insufficient memory and limited computing power of the current vehicle-mounted embedded device. The driver’s PERCLOS and frequency of open mouth (FOM) were used to realize the judgment of the driver’s fatigue state. The real-time image segmentation and drowsiness using machine learning methodologies were implemented by Altameem et al. [18], and an emotion detection method based on SVM was implemented using facial expressions. You et al. [19] proposed a real-time driving drowsiness detection algorithm that considers the individual differences of the driver, a new parameter, called EAR, was introduced to evaluate the drowsiness of the driver in the current frame, and a deep cascaded convolutional neural network was constructed to detect the face region, which avoids the problem of poor accuracy caused by artificial feature extraction. A fully automated driver fatigue status detection algorithm using driving images was proposed by Zhao et al. [2]. The multitask convolutional neural network (MTCNN) architecture was employed in face detection and feature point location, and the region of interest (ROI) was extracted using feature points. The convolutional neural network, named EM-CNN, was used to detect the states of the eyes and mouth from the ROI images. Similarly, Chen et al. [4] used MTCNN to detect a human face: an open-source software library (DLIB) was used to locate facial landmarks to extract the fatigue feature vector of each frame, and the long short-term memory (LSTM) network was used to obtain a final fatigue feature value. So, it can be seen from the literatures mentioned above that in all the features, the characteristics of the eyes and the mouth are the most widely used, and the establishment of complex models and the large amount of data to be processed pose new challenges to the computing power of computers. More relative references can also be founded [20,21,22,23,24,25,26].

### 2.3. Detection Based on Multi-Information Fusion

In the paper of Mühlbacher-Karrer et al. [27], a driver state detection system based on cellular neural networks (CNNs) to monitor the driver’s stress level was presented, which combines the input of both physiological sensors and a novel capacitive touch and position detection sensor. Threefold cross-validation was applied to evaluate this concept. In the study of Sun et al. [3], the fatigue driving state was estimated based on two categories of measurements: driver facial expression and vehicle operating condition. In addition, RGB-D cameras are increasingly used for facial feature recognition; their wide application may bring revolutionary changes to information fusion technology. In particular, for what concerns face morphology, 3D has led to the possibility of obtaining face depth maps highly close to reality and consequently an improvement of the starting point for further analysis such as face detection, face identification, and facial expression recognition [28]. Another very big advantage of the RGB-D camera is that it can directly obtain information on the human heart rate. Some relative works have already been investigated, for example, a single RGB-D camera was used by Du et al. [29] to extract three fatigue features: heart rate, eye openness level, and mouth openness level. A novel multimodal fusion recurrent neural network (MFRNN) integrating the three features was proposed to improve the accuracy of driver fatigue detection.

## 3. Methods

In this paper, the sample diversity should be made as much as possible when establishing the face sample dataset, because, in the actual driving process, the face image is affected by factors such as the light environment, facial expressions, and driver posture, as well as some factors related to individual differences, such as different gender, age, skin color, and so on. Different datasets were used in different sections.

For the dataset used for training the model, 2500 samples from the YawDD dataset [30], LFPW dataset [31], and self-built dataset were used to train the detector. The size of which was adjusted to 400 × 300. For face detection, datasets were labeled with Python’s own LabelImg tool [32]. For face landmarks detection, each image was marked with 68 key points; the marked coordinate information was saved in a file for training. In addition, 800 samples from YawDD and a self-built dataset with a size of 400 × 300 were used to evaluate the effectiveness of landmark detection.

The experimental dataset used for fatigue labeling and fatigue detection was collected by Rmoncam G500, the number of samples is 500, which includes 150 awake samples and 350 fatigue samples, and the size of the image is set to 640 × 480.

The whole process of driver fatigue detection in this paper can be divided into five steps: face detection and tracking, detection of facial landmarks, selection of three typical fatigue features, the judgment of fatigue state based on the BP neural network model, and fatigue judgment based on the time cumulative effect model.

### 3.1. Face Detection and Tracking

In 2001, Viola and Jones [33,34] combined the Adaboost detection algorithm [35] with a cascade algorithm to construct a new detector. This algorithm has relatively higher detection speed and accuracy, so it is widely used today.

This method uses the same training sample set for iterative training to obtain different weak classifiers. In each training, the weight of the weak classifier and each sample input in the next training is determined according to the accuracy of the current classification. The weight of correctly classified samples is reduced in the next training, the weight of incorrectly classified samples is increased, and the training will continue until reaching the set maximum number of iterations or the specified error rate. Finally, the strong classifiers were built by combining these weak classifiers.

In general, for a single strong classifier, the identification accuracy can be improved by increasing the number of weak classifiers, which also increases the complexity of the calculation. Viola et al. [33,34] proposed a cascade approach to increase detection speed and reduce false detection rate. Cascade connect multiple strong classifiers in series with the following steps: sorting all strong classifiers according to their complexity, for an input image window, it is firstly detected by the simplest strong classifier, if the detection result is not-face, it will be directly removed, otherwise, it enters the next strong classifier detection. This detection continues until the last classifier. With the increasing complexity of the classifier, the number of image windows to be detected decreases; this method can greatly improve the detection accuracy and speed.

In the process of face detection, the position and size of the face in different images are changing. To detect faces in different positions, it is necessary to slide the detection window or carry out a multi-scale transformation. The first step is to determine the size of the original detection window and then move this window to the sub-image until all regions are covered. Usually, if one area is detected as a face, its nearby multiple overlapping areas will also be detected as a face. Before obtaining the result of face detection, it is also necessary to merge the repeatedly detected faces.

During the face detection process, if the face is blocked by occluded objects or the driver turns around, detection failure may occur. For these situations, the Kalman filter algorithm [36] can be used for face movement prediction. When the occlusion is encountered or the confidence of the detected frame does not reach the set threshold, the Kalman filter algorithm was used to track and predict the coordinates of the upper left and lower right corners of the face frame. Even without using the Kalman filter, the work of face detection can still be carried out, but the introduction of the Kalman filter significantly enhances the stability of target tracking.

### 3.2. Facial Landmark Detection Based on Cascade Regression Tree

The ensemble of regression trees (ERT) method proposed by Kazemi et al. [37] was adopted to detect the facial landmarks. The whole process of this method includes a cascade of regressors, learning each regressor in the cascade, shape invariant spit tests, choosing the node spilt, feature selection, and handling missing labels.

In this method, a cascade gradient boosting decision trees (GBDT) algorithm was established to detect the face from the initial shape to the real shape step by step, which is the core principle of the proposed ERT method. Each leaf node of each GBDT stores a residual regression. When the input falls on a node, the residual is added to the input for the purpose of regression. Finally, all the residuals are superimposed together to achieve the purpose of face alignment. A more detailed explanation of this method can be found in the paper mentioned above. The GBDT method has two main characteristics: (1) the relationship between different trees is serial rather than parallel, and the latter tree is built based on the former tree; (2) the leaf nodes of each tree store residuals and only through the residuals saved on the leaf nodes can the shape be continuously returned to the real shape.

### 3.3. Selection of the Key Point Features

After facial landmark detection, the Adaboost algorithm was used to realize face recognition, and the landmarks describing the outline of the human eye or mouth were detected using the ERT algorithm. Then, the characteristics of the eyes or mouth can be further analyzed.

#### 3.3.1. Definition of Eye Aspect Ratio

**Figure 1:**
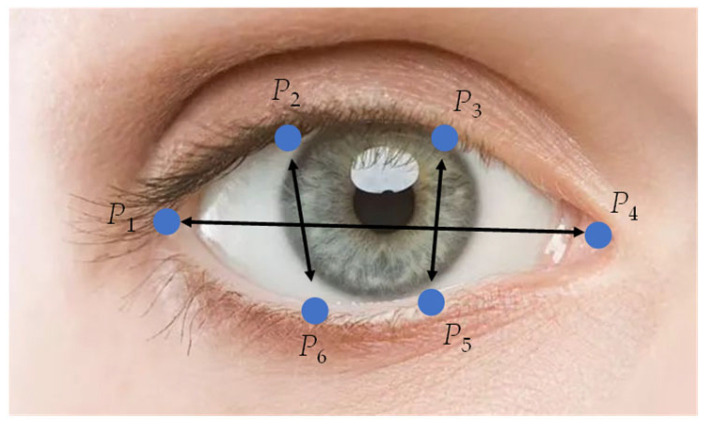
Key points selected around the eye for calculating EAR.

EAR is commonly used to calculate the state of eye opening and closing [19]. Generally, when the eye is open, the ratio of width to length is a fixed value. When the eye is closed, the length of the eye remains the same; the aspect ratio changes rapidly when the width decreases. The selected feature points around the eyes are shown in Figure 1. The calculation formula of EAR can be written as:

(1) $$\mathrm{EAR}=\frac{\left\|P_{2}-P_{6}\right\|+\left\|P_{3}-P_{5}\right\|}{2\left\|P_{1}-P_{4}\right\|}$$

in which *P*_1_~*P*_6_ represent the coordinates of feature points around the eye. The six landmarks are found to be inaccurate to varying degrees, especially in conditions of backlight or dim, the eyes of the person tested being closed, or they have deeper eye sockets, and so on.

Therefore, an improved method combining key point coordinates with image processing was used to calculate EAR. The main steps are as follows: (1) obtaining the coordinates of the feature points *P*_2_, *P*_3_, *P*_5_, and *P*_6_, receiving the enclosing rectangle formed by these four points, extending the height of the rectangle with 50% both in the up and down directions, and intercepting the image in the rectangle, by which, the obtained image will not be too large or too small, the contrast between eye features and non-eye features is more obvious, so the changes in eye features are much easier detect; (2) image graying, filtering, and denoising, as well as the Otsu binarization [38], were carried out on the image to make sure that the image only contained the eye region; (3) feature points and lines were used to calculate the angle of eye tilt and the eyes in the image were horizontally corrected; (4) calculating the sum of gray values of each column in the captured image, setting its maximum value as *M*, the height of the eye can be expressed as: *H* = *M*/255; (5) the eye width *W* is defined as the straight line distance between feature points *P*_1_ and *P*_4_; (6) calculating EAR by function EAR = *H*/*W*.

**Figure 2:**
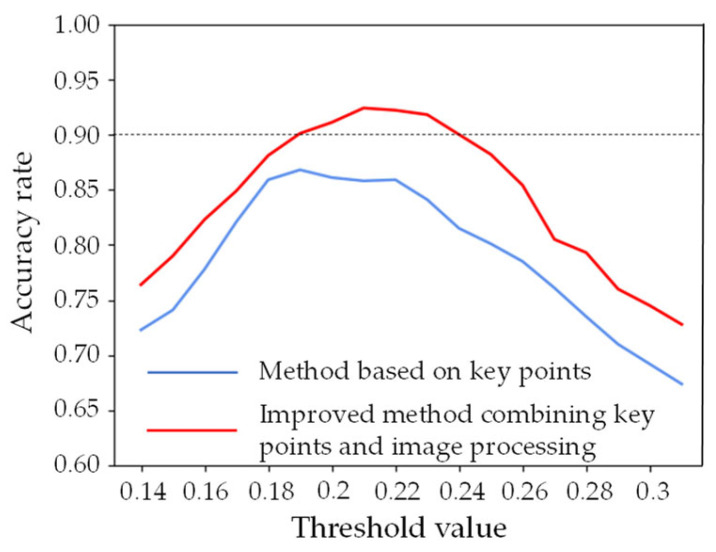
Detection results of open and closed eyes using two different methods.

In this paper, a test dataset was built to verify the effectiveness of the improved method mentioned above. The total number of images is 800, with 400 open eyes and 400 closed eyes. The size is adjusted to 400 × 300. The detection results of open and closed eyes are shown in Figure 2.

It can be seen from Figure 2 that the detection accuracy of the improved method proposed in this paper is significantly higher than that of the traditional key points-based method. The maximum accuracy of the improved model is about 92.4%, which appears when the threshold is about 0.21. So, the value of EAR is finally defined as 0.21, which means when EAR > 0.21, the eyes are considered open; otherwise, they are closed.

#### 3.3.2. Definition of Mouth Aspect Ratio

**Figure 3:**
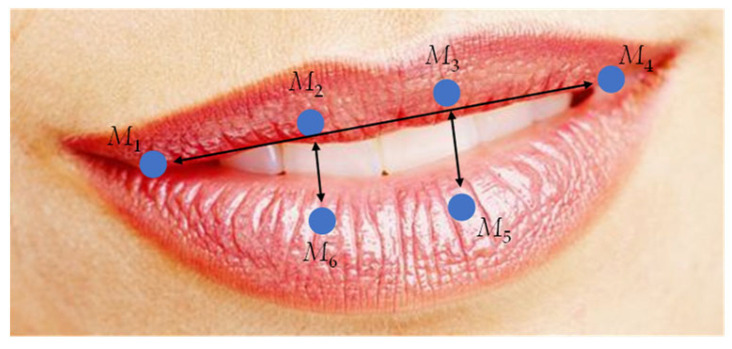
Key points selected around the mouth for calculating MAR.

Similarly, in addition to the changes in eye features, the changes in mouth features are also closely related to fatigue state. Generally, when a person is in a state of fatigue, the behavior of yawning will increase. Usually, the mouth aspect ratio (MAR) or the frequency of open mouth (FOM) [17] is used to measure the degree of opening and closing of the mouth. Mouth feature points *M*_1_~*M*_6_ are shown in Figure 3.

The equation of MAR can be written as:

(2) $$\mathrm{MAR}=\frac{\left\|M_{2}-M_{6}\right\|+\left\|M_{3}-M_{5}\right\|}{2\left\|M_{1}-M_{4}\right\|}$$

in which *M*_1_~*M*_6_ represent the coordinates of selected feature points around the mouth.

The key points-based method was used to calculate MAR. The determination of the threshold value of MAR is similar to the EAR. When MAR = 0.4, the maximum accuracy is about 99.5%, so 0.4 was selected as the threshold value for determining whether the mouth is open or closed.

### 3.4. Selection of the Fatigue Features

In this paper, three typical indicators—the longest period of continuous eye closure, number of yawns, and PERCLOS—were selected to train the corresponding models. The values of which can be calculated according to the states of eye and mouth judged by EAR and MAR.

#### 3.4.1. Longest Period of Continuous Eye Closure

When a driver is in a fatigue state, the duration of eye closure becomes longer with the increase in fatigue degree. The continuous eye closing time can be calculated by counting the number of consecutive frames in which the EAR value is less than the previously calculated threshold of 0.21. When the driver’s eyes start to close, the number of consecutive closed frames can be counted; the continuous closed time *F_t_* is defined as [20]:

(3) $$F_{t}=(N_{end}-N_{start})\cdot T_{0}$$

in which *N_start_* is the sequence number of the eyes beginning to close in the video frame, *N_end_* is the sequence number of the eyes in the video frame from the continuously closed state to the open state, and *T*_0_ represents the time interval of each frame image.

#### 3.4.2. Number of Yawns

Normally, when a person yawns, the mouth will open wide and keep the state of opening for a few seconds. The state of mouth opening or closing can be judged by the previously calculated threshold value of 0.4. Some facial expressions, such as yawning, speaking, and laughing, can make the mouth open. However, under normal circumstances, yawns usually last for approximately 6 s. In the case of talking, laughing, and so on, the mouth only opens for a short time. Thus, this study defines yawning by counting the duration of mouth opening, as defined below [20]:

(4) $$F_{m}=(M_{end}-M_{start})\cdot T_{0}$$

in which *M_start_* is the sequence number of the mouth beginning to close in the video frame, *M_end_* is the sequence number of the mouth in the video frame from the continuously closed state to the open state, and *T*_0_ represents the time interval of each frame image. By this method, the number of yawns can be counted.

#### 3.4.3. PERCLOS

**Figure 4:**
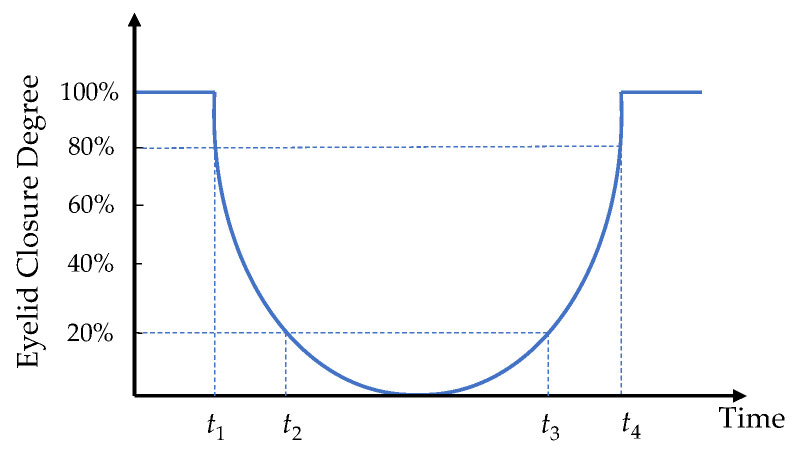
Schematic diagram of measuring PERCLOS.

PERCLOS is the percentage of eyelid closure over the pupil over time and reflects slow eyelid closures (“droops”) rather than blinks. PERCLOS is considered the most promising known real-time measure of alertness for in-vehicle drowsiness detection systems. A PERCLOS drowsiness metric was firstly established by Wierwille et al. [39] in a driving simulator study as the proportion of time in a minute that the eyes are at least 80 percent closed. The calculation formula can be written as:

(5) $$\mathrm{PERCLOS}=\frac{t_{3}-t_{2}}{t_{4}-t_{1}}\times 100\%$$

where the PERCLOS is the value of PERCLOS, *t*_1_ and *t*_2_ are the time that the eyes are closed from the largest to 80% and 80% to 20%, *t*_3_ is the time from 20% closed to 20% open, and *t*_4_ is the amount of time spent when eyes are open from 20% to 80%. The schematic diagram of this method is shown in Figure 4.

In an actual driving situation, the video stream is converted into a frame image by the camera acquisition device for processing, so the PERCLOS method can also be defined as the ratio of the number of closed frames per unit time to the total number of frames, that is [20]:

(6) $$F_{p}=\frac{\sum_{i=1}^{N}f_{i}}{N}\times 100\%$$

where *F_p_* represents the PERCLOS value, *N* represents the total number of frames of video per unit time, and the numerator part represents the number of closed eyes per unit time.

### 3.5. Driver Fatigue Detection Model Based on BP Neural Network Model

Multi-feature fusion modeling is a process that integrates various information, including processes such as data pre-processing, accuracy matching, prediction or estimation, decision making, and so on, finally, obtaining a more reliable and accurate decision than the method based on a single information source. In this paper, Back Propagation (BP) neural network was used as the feature fusion algorithm.

BP neural network is an algorithm proposed by Rumelhart et al. [40] in 1985. A typical BP neural network consists of an input layer, hidden layer, and output layer. In this paper, the implicit relationship between three facial features and fatigue was built through neural network training. The data used for training were introduced at the beginning of Section 3. In total, 90% of the data was used as a training set and 10% of the data was used as a verification set.

Considering the training efficiency and comprehensive application scenarios of the network, the hidden layer selected was 1. The input parameters are the longest continuous eye closing time, the number of yawns, and PERCLOS values; the number of nodes in the input layer was 3. The output item is whether the current sample is fatigued; the number of nodes in the output layer was 1. Besides, the cross-entropy loss function was used as the loss function, the batch size was 8, and the epoch was set to 4000. Adam optimization algorithm was selected as the optimizer, which can calculate the adaptive learning rate of each parameter, and its convergence speed is faster and the learning effect is more effective.

**Figure 5:**
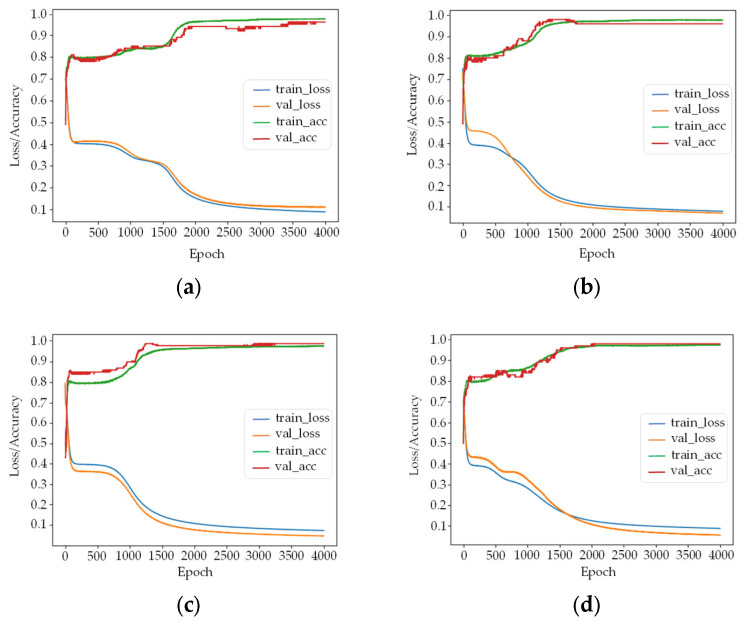

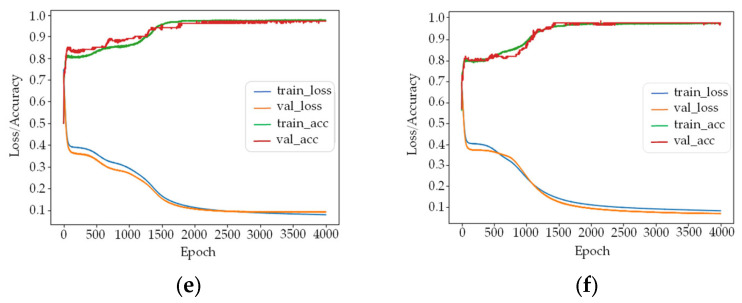
Training loss and accuracy of selecting different node numbers in the hidden layer of the BP neural network model. Figures (**a**–**f**) represent the training results of the model when the number of hidden nodes is 3–8, respectively.

Until now, there is a lack of reliable theory to guide the determination of the node number of hidden layers in a neural network. For the neural network structure with only one hidden layer, the number of nodes represents the nonlinear degree of the network. When the number of hidden layer nodes is too small, the generalization ability of the network is poor. When there are too many nodes in the hidden layer, the training time increases greatly, and the training results may not be reliable. Therefore, it is necessary to determine the appropriate number of hidden layers by conducting multiple tests according to the empirical formula. The most used empirical formula adopted for selecting the number of hidden layer nodes is shown in Equation (7).

(7) $$m=\sqrt{i+j}+c$$

in which *m* represents the number of hidden layers, *i* represents the number of nodes in the input layer, *j* represents the number of nodes in the output layer, and *c* is a constant between 1 and 10. According to the empirical formula mentioned above, the hidden nodes from 3 to 8 were selected in this paper for comparison. The calculation results are shown in Figure 5.

In Figure 5a–f represent the training loss and accuracy results of the model when the number of hidden nodes is 3–8, respectively. When the number of nodes is 3, 5, and 6, the difference between the loss of training and verification sets is between 0.02 and 0.03, which is larger than nodes 4, 7, and 8. The accuracy of training and verification sets of these training models are in the range of 0.97~0.99. Considering the complexity of the network model and training effect, the number of nodes of the hidden layer was finally set as 4.

### 3.6. Driver Fatigue Detection Model Based on Time Accumulation Effect

Fatigue refers to a kind of phenomenon where the organism’s state drops obviously after continuous work or study. In other words, fatigue is a process of accumulation and will continue to be maintained after entering the fatigue state. At present, most scholars only study the fatigue state of the samples in a certain time window, and the relationship between the fatigue state and time accumulation is ignored.

In the process of fatigue detection, some noise information exists in video samples; these noises are generated due to the changes in the facial expression such as smiling, speaking, and so on. Because of the noise information, even if the subjects are in the waking state, sometimes it will also be judged into a fatigued state. Aiming at this problem, a time accumulative fatigue detection model was proposed to reflect the cumulative effect of fatigue over time and eliminate the influence of the noise information.

Since the generation of noise information is stochastic, the established model should be able to filter out the random noise information in the samples while maintaining a high detection rate. According to this characteristic, this paper proposes a fatigue detection model by defining a cumulative value of fatigue *a_F_*, which can be written as:

(8) $$a_{F}=\frac{1}{n}\sum_{i=1}^{n}\left[x_{i}\times \mathrm{sigmoid}\;(x_{i})-(1-x_{i})\times \mathrm{sigmoid}\;(x_{i})\right]$$

where *x_i_* is a 0–1 variable, representing the two states of the current segment. 0 represents the awake state, and 1 represents the fatigue state. *n* is the number of segments divided and *n* > 1. The Sigmoid function, commonly used in logistic regression problems, is a smooth, strictly monotone increasing function with a range of 0 to 1. The purpose of introducing the Sigmoid function is to keep the value of the output fatigue degree between –1 and 1.

(9) $$\mathrm{sigmoid}\;(x_{i})=1/\left(1-e^{-x_{i}}\right)$$

When the input value of a segment is 0, this means the input is awake. The first half part of Equation (8) is 0, and the overall output is a negative number. Thus, the fatigue accumulation model will reduce the corresponding value. When the input of a segment is 1, this is a fatigue state. The latter half part of Equation (8) is 0, and the overall output is a positive number. Thus, the fatigue accumulation model will increase the corresponding value.

After calculating all segments, if the accumulated fatigue value *a_F_* is greater than the threshold value, the sample is judged to be in the fatigue state; otherwise, it is in the awake state. In the actual calculation process, when a sample is divided into *n* segments, there will be *n* + 1 different fatigue cumulative values, and the calculation expression can be written as:

(10) $$a_{F}^{k}=\frac{1}{n}[k\times \mathrm{sigmoid}\;(1)-(n-k)\times \mathrm{sigmoid}\;(0)]\;\;\;\;k=0,1,\ldots ,n$$

$a_{F}^{k}$ indicates when the sample is divided into *n* segments, *k* segmented samples are detected as fatigue. The threshold value *θ_F_* is defined as the average value of two adjacent cumulative values. The calculation formula is shown as:

(11) $$\theta_{F}=\frac{a_{F}^{k}+a_{F}^{k+1}}{2}\;\;\;\;k=0,1,\ldots ,n$$

To compare the results of the proposed model with that of the BP neural network model, the dataset and basic parameters used for training the time accumulation effect model were set as the same as the BP neural network model. The proposed time accumulation effect model is a general model; the segment number *n* can be determined according to the length of the selected video time window and the usual duration of the extracted feature. The best threshold value of *θ_F_* can be determined by the fatigue detection accuracy, which is an empirical value obtained by comparing the detection results between different threshold values. The determination of these parameters will be detailed and described with an example in the following section.

## 4. Results

According to the model established above, face detection and state judgment of the eyes and mouth were carried out, with three typical features selected. In this section, the fatigue state of the driver will be judged based on the BP neural network model and the time accumulation effect model, respectively, and the results will also be compared.

### 4.1. Results of Fatigue Determination Based on the BP Neural Network Model

**Figure 6:**
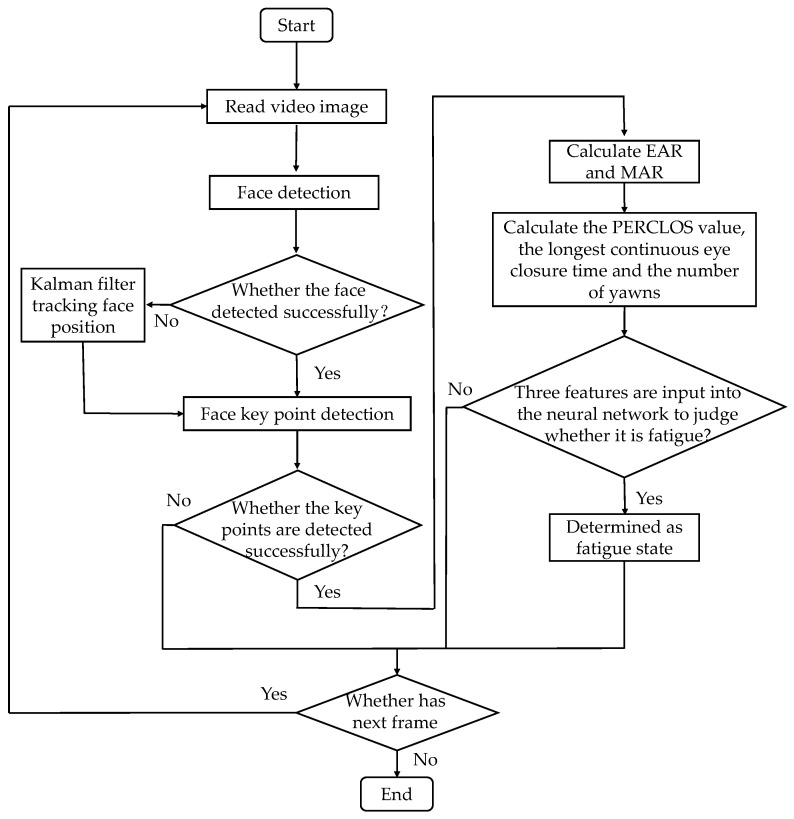
Flow chart of fatigue driving detection based on the BP neural network model.

The steps of fatigue detection based on the BP neural network model are shown in Figure 6. First of all, the Adaboost algorithm was used to detect the face and its landmarks. In the case of situations such as the face being obscured, the Kalman filter method was used to trace the face. Then, EAR and MAR were calculated according to the coordinate information of the landmarks. Finally, the longest continuous eye closing time, the number of yawns, and PERCLOS were input into the neural network for fatigue detection.

In order to verify the accuracy and robustness of the algorithm, this paper designs two different driving scenarios: Scenario 1: subjects keep the normal simulated driving state and no changes in facial expressions, such as smiling or speaking, appear on their faces; Scenario 2: during simulated driving, the subjects produce changes in facial expressions, such as smiling or speaking. For a traditional neural network model or relevant machine learning algorithm, through the comparison of these two scenarios, we can obviously see the incorrect detections caused by the facial expressions.

Experimental data was recorded at the driving simulation platform with a Rmoncam G500 camera installed on the front of the driver. A total of 7 subjects were divided into two groups: the first group of 4 subjects simulated driving conditions according to scenario 1, and the second group of 3 subjects simulated driving conditions according to scenario 2. All driving conditions were carried out under the condition of sufficient sunlight, and the video data was segmented according to the time window of 60 s. Both scenarios contain samples of fatigue and awake. Finally, 15 fatigue samples and 45 awake samples were selected for each scenario. The composition of sample data is explained in Table 1.

For two different scenarios, a total of 120 samples were used for fatigue detection calculation. The detection results using the BP neural network are shown in Table 2 and Table 3.

The experimental results of scenario 1 show that the detection accuracy is 88.9% in the fatigue state and 93.3% in the fatigue state, and the overall detection accuracy is 90%, which is highly accurate and reliable.

The experimental detection results of scenario 2 show that the detection accuracy rate under the awake state is 75.6%, which is 13.3% lower than the detection accuracy rate under the driving state of scenario 1. The detection accuracy under the fatigue state is 86.7%, and the overall detection accuracy is 78.3%.

By analyzing the detected samples of wrong results, it can be found that the EAR value is reduced while the subjects are smiling or talking. The EAR drops below the threshold value and lasts for several seconds, which will cause the eigenvalues of the PERCLOS and the longest continuous eye closing time to increase. So, the changes in facial expressions will lead to incorrect detection, which should be further explored, and the influence of this noise information should be eliminated.

### 4.2. Results Based on the Time Cumulative Effect Model

**Figure 7:**
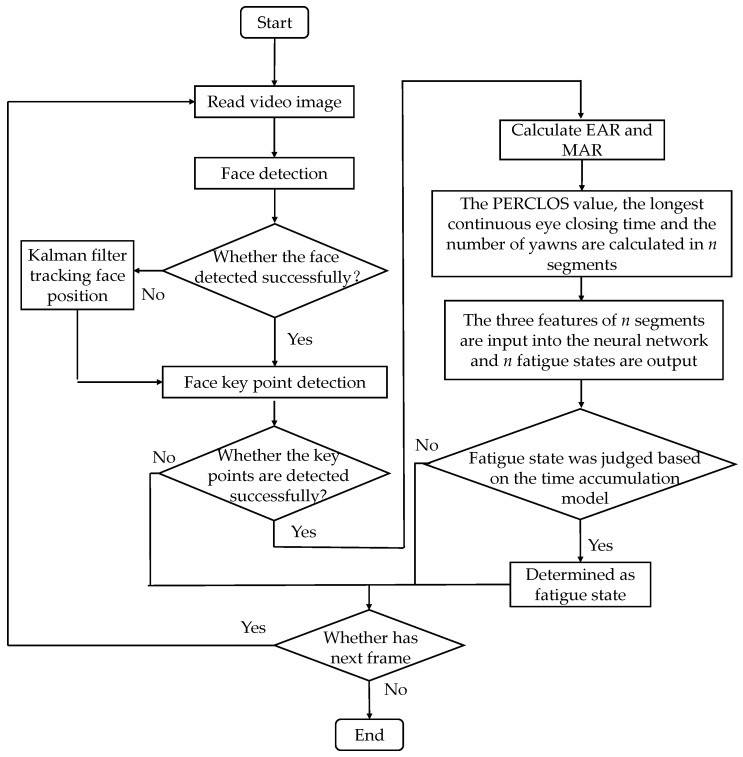
Flow chart of fatigue driving detection based on the time cumulative effect model.

A flow chart of driver fatigue detection based on the time accumulation effect is shown in Figure 7. Compared with the traditional BP neural network, after obtaining the EAR and MAR values, the experimental samples (e.g., the time window is 60 s or 120 s) need to be further segmented. Each video sample was further divided into *n* segments, and the three eigenvalues of the longest continuous eye closing time and the number of yawns and PERCLOS were calculated, respectively. The three eigenvalues of *n* segments were re-input into the neural network to obtain *n* fatigue states. Finally, the final fatigue states of the samples can be determined based on the time accumulation model.

Short and long video experiments were designed in this section to verify the accuracy and effectiveness of the algorithm.

#### 4.2.1. Fatigue Test Results of Short Video Samples

According to the proposed time cumulative effect model, generally, the more segments divided in each time window, the more accurate the judgment of the fatigue state. The determination of segment number is mainly influenced by two factors: the length of the time window and the duration of each extracted typical feature. For example, when the time window is set to 60 s, the average duration of yawn usually lasts about 6 s. In this case, the value of the number of segments should not exceed 10; otherwise, the characteristic of a yawn cannot be acquired completely. Moreover, a sufficient margin should be left. The time windows defined in this paper are 60 s and 120 s. In order to take both situations into account, the segment number can be 3, …, and so on. In this paper, the method is introduced only when the segment number is 3. The comparison with other values and the improvement of the model will be the focus of future research.

The optimal threshold should be determined before fatigue detection by using the time cumulative effect model. Different thresholds were used to judge the fatigue of the same test sample, and the threshold with the highest detection accuracy was selected as the optimal threshold. In this paper, the number of segments *n* = 3 was taken as an example to detect the fatigue state and select the optimal threshold. In the case of *n* = 3, the cumulative fatigue values were calculated according to Equation (10); the calculation results are −0.5, −0.089, 0.321, and 0.731. The average value of two adjacent cumulative values was calculated according to Equation (11); the results are −0.295, 0.116, and 0.526, respectively. Then, the fatigue detection of 500 experimental samples was implemented based on a combination of the neural network and time cumulative effect model, and the detection accuracy of the different threshold values is shown in Table 4.

It can be seen from the above table that when the threshold value is 0.116, the fatigue detection accuracy is the highest. Therefore, the fatigue threshold was set as 0.116 in this paper. When the threshold is smaller than 0.116, the sample is regarded as in the awake state; otherwise, it is in the fatigue state. This method was used to perform fatigue detection again for the samples of scenarios 1 and 2, with a time window of 60 s and a size of 640 × 480 for each sample. The detection results are shown in Table 5 and Table 6.

It can be seen from Table 5 and Table 6 that the fatigue detection results based on the BP neural network are apparently affected by the noise information in the detection process, but the influence of which can be effectively eliminated by the proposed time accumulation model.

#### 4.2.2. Fatigue Test Results of Long Video Samples

Although the short video sample has been used to verify the accuracy of the algorithm, it does not reflect the stability of the algorithm in a continuous video sample, and it cannot detect the exact time from the awake state to the fatigue state.

**Figure 8:**
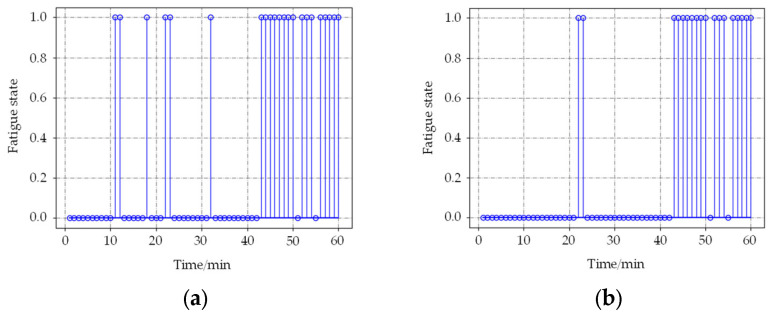
Detection results of the two methods when the time window is 60 s. (**a**) shows results based on the BP neural network; (**b**) shows results based on the time accumulation model.

**Figure 9:**
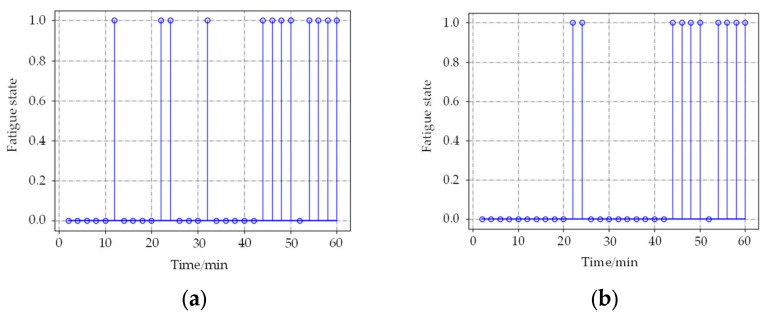
Detection results of the two methods when the time window is 120 s. (**a**) shows results based on the BP neural network; (**b**) shows results based on the time accumulation model.

For these problems, a long video sample detection experiment was designed. In order to test whether the noise information can be filtered by using the proposed method, subjects made some random facial expressions to generate some noise information when they were awake. The duration of the video sample collected was 1 h. The time windows were set to 60 s and 120 s, and the detection results based on the neural network, as well as the time accumulation model, were compared. The detection results are shown in Figure 8 and Figure 9.

In Figure 8 and Figure 9, 0 represents the awake state detected by the algorithm, and 1 represents the fatigue state. When the time window is 60 s, the results based on the BP neural network produce 6 incorrect detections in the awake state at 11, 18, 22, and 32 min, respectively. The improved method generates only twice. When the time window is 120 s, the detection result is similar to 60 s. It indicates that the method based on the time accumulation effect model can effectively filter the noise information and improve the detection accuracy. About judging the exact time from awake to fatigue state, the results are almost the same; the state of the subject changed from awake to the fatigue state at about 42 min, which is in accordance with the actual situation.

To sum up, the proposed fatigue detection method effectively combines the neural network with the time accumulation effect, and achieves better detection results, which is a reliable method for fatigue driving detection.

## 5. Discussion

For the BP neural network model, a comparative study on two different experimental scenarios was conducted. According to the results of the two scenes, it can be clearly seen that when using traditional methods to distinguish fatigue, factors such as the subjects’ smiling and speaking will have a significant impact on the detection of fatigue state. The consideration of these factors is of great significance. In different conditions with and without these factors, the model’s judgment accuracy is greatly different in both fatigue and awake states.

In the awake state, the accuracy of prediction without and with interference factors is 88.9% and 75.6%, respectively; the accuracy of prediction is reduced by 13.3%. In the fatigue state, the accuracy under conditions without and with interference factors are 93.3% and 86.7%, respectively; the existence of interference factors reduces the accuracy of prediction by 6.6%. For the total accuracy rate, the corresponding values are 90% and 78.3%, respectively; the existence of interference factors reduces the total accuracy rate by 11.9%. Therefore, changes in facial expressions have a great impact on the results of fatigue state judgment, and further research is needed.

For the above problems, the fatigue detection results of the BP neural network model and the proposed time cumulative effect model were compared with both short and long video samples.

In the case of the short video, by using the proposed time cumulative effect model, the overall judgment accuracy of the fatigue state in scenario 1 is improved from 90% of the BP neural network model to 93.3%, an increase of 3.3%. In scenario 2, the total judgment accuracy increases from 78.3% to 86.7%, an increase of 8.4%. For the fatigue judgment of long video, the case of incorrect detection is significantly reduced. When the time window is 60 s, the number of incorrect detections in the awake state decreased from 6 times in the BP neural network model to 2 times; when the time window is set to 120 s, the number of incorrect detections decreases from 4 to 2 times. So, the model proposed in this paper has a good capability of eliminating the incorrect detection of fatigue state. For short time windows, the model proposed in this paper can well reflect the cumulative effect of fatigue in time. Subsequent studies should focus on the establishment of models of sustained fatigue accumulation over a longer time span.

## 6. Conclusions

In this paper, a fatigue detection algorithm based on the time accumulation effect was proposed to detect the state changes of drivers. The main conclusions are as follows.

First of all, aiming at the problem of large calculation error of EAR, an improved method based on key points and image processing was adopted to improve the judgment accuracy of eye opening and closing. The face was further traced by using the Kalman filter method. By which, the excessive EAR error caused by the dim light, deeper eye sockets, head movement, and so on was effectively reduced.

Then, for the traditional BP neural network model, the changes in facial expressions such as smiling and speaking will lead to incorrect detection. A model based on time cumulative effect was proposed to eliminate the influence of incorrect detection caused by the noise information. Compared with the traditional BP neural network model, the accuracy of the prediction results has been significantly improved in different scenarios with better robustness. At the same time, the method presented in this paper does not significantly increase the demand for data processing.

Although the model proposed in this paper has high accuracy and reliability, there are still some limitations: (1) the model proposed in this paper has proved its superiority over the BP neural network model, but there is no comparison with other neural network models or machine learning models, meaning the improvement of prediction accuracy of other types of models should be further studied; (2) for the segmented short videos, this model well reflects the cumulative effect of time, which can be further optimized and a more general model should be considered and built for any general videos; (3) this paper focuses on the analysis based on video data, application of depth camera was not considered, fatigue detection based on depth camera is also an important aspect; (4) The fusion of multi-information is only considered for different image features, how to combine different types of data and conduct a comprehensive analysis of different data will also be an urgent issue in the future.

## Figures and Tables

**Table 1:** Composition of test dataset used for the BP neural network model.

Total Number of Samples	Scenario 1		Scenario 2	
	Number of Fatigue Samples	Number of Awake Samples	Number of Fatigue Samples	Number of Awake Samples
120	15	45	15	45

**Table 2:** Fatigue test results of scenario 1 according to the BP neural network model.

Actual State of the Sample	Number of Samples	Correct Number	Accuracy
Awake state	45	40	88.9%
Fatigue state	15	14	93.3%
Total number	60	54	90%

**Table 3:** Fatigue test results of scenario 2 according to the BP neural network model.

Actual State of the Sample	Number of Samples	Correct Number	Accuracy
Awake state	45	34	75.6%
Fatigue state	15	13	86.7%
Total number	60	47	78.3%

**Table 4:** Fatigue detection accuracy of different thresholds in the case of segment number *n* = 3 by using the time cumulative effect model.

**Threshold**	–0.295	0.116	0.526
Number of Correct Detections	462	478	469
Accuracy	92.4%	95.6%	87.8%

**Table 5:** Comparisons of fatigue detection results of the BP neural network model and the proposed time accumulation model for scenario 1.

Method	Correct Number of Awake State	Correct Number of Fatigue State	Total Correct Number	Accuracy
BP neural network based	40	14	54	90.0%
Time cumulative effect based	42	14	56	93.3%

**Table 6:** Comparisons of fatigue detection results of the BP neural network model and the proposed time accumulation model for scenario 2.

Method	Correct Number of Awake State	Correct Number of Fatigue State	Total Correct Number	Accuracy
BP neural network based	34	13	47	78.3%
Time cumulative effect based	39	13	52	86.7%

## Data Availability

The data presented in this study are available on request from the corresponding author.
